# Antimicrobial resistance in the wild: Insights from epigenetics

**DOI:** 10.1111/eva.13707

**Published:** 2024-05-29

**Authors:** Mariana Villalba de la Peña, Ilkka Kronholm

**Affiliations:** ^1^ Department of Biological and Environmental Science University of Jyväskylä Jyväskylä Finland

**Keywords:** adaptation, antimicrobial resistance, epigenetics, microbes, natural environment

## Abstract

Spreading of bacterial and fungal strains that are resistant to antimicrobials poses a serious threat to the well‐being of humans, animals, and plants. Antimicrobial resistance has been mainly investigated in clinical settings. However, throughout their evolutionary history microorganisms in the wild have encountered antimicrobial substances, forcing them to evolve strategies to combat antimicrobial action. It is well known that many of these strategies are based on genetic mechanisms, but these do not fully explain important aspects of the antimicrobial response such as the rapid development of resistance, reversible phenotypes, and hetero‐resistance. Consequently, attention has turned toward epigenetic pathways that may offer additional insights into antimicrobial mechanisms. The aim of this review is to explore the epigenetic mechanisms that confer antimicrobial resistance, focusing on those that might be relevant for resistance in the wild. First, we examine the presence of antimicrobials in natural settings. Then we describe the documented epigenetic mechanisms in bacteria and fungi associated with antimicrobial resistance and discuss innovative epigenetic editing techniques to establish causality in this context. Finally, we discuss the relevance of these epigenetic mechanisms on the evolutionary dynamics of antimicrobial resistance in the wild, emphasizing the critical role of priming in the adaptation process. We underscore the necessity of incorporating non‐genetic mechanisms into our understanding of antimicrobial resistance evolution. These mechanisms offer invaluable insights into the dynamics of antimicrobial adaptation within natural ecosystems.

## 1 INTRODUCTION

The discovery and widespread availability of antimicrobials have greatly improved the treatment of bacterial and fungal infections in the modern era. However, their excessive use in various sectors, including agriculture, livestock farming, and human medicine, has resulted in a rapid increase in antimicrobial resistance (AMR) as the result of the strong selection pressure that antimicrobial substances impose on microbes. This rapid proliferation of AMR poses a severe threat to the well‐being of humans, animals, and plants (Fisher et al., 2022; Frieri et al., 2017; Low & Rotstein, 2011; Murray et al., 2022). Note that we use the term antibiotics to refer to substances that either kill or inhibit the growth of bacteria, while antifungals denotes substances targeting fungi. The broader term antimicrobial encompasses both antibiotics and antifungals.

Given the pressing problem that AMR represents, considerable research efforts have been directed toward elucidating the mechanisms behind AMR in clinical settings. However, a significant knowledge gap still exists when it comes to understanding antimicrobial resistance in natural environments. This is crucial as wild‐resistant microbial populations serve as environmental reservoirs that can transfer to human populations, impact the persistence of wild animals and plants, and profoundly affect environmental health (Polianciuc et al., 2020). Several fungal and bacterial epidemics in the wild show the great risk that pathogenic microbes represent for biodiversity and environmental health (for example, see Cheng et al., 2011; Espelund & Klaveness, 2014; Fisher et al., 2009, 2012; Frick et al., 2010; Sandmeier et al., 2009). Wildlife epidemics can be intensified by human activities due to the introduction of alien pathogens (Fisher et al., 2012). However, in wildlife, the health consequences of the antimicrobial‐resistant strains or antimicrobial treatments remain poorly understood (Arnold et al., 2016).

Mutation and horizontal transfer of resistance genes have been very well described and are traditionally considered the main mechanisms through which microbes evolve resistance (Hiltunen et al., 2017). However, new evidence suggests that resistance can also evolve through alternative non‐genetic routes, such as epigenetic mechanisms (Sabarís et al., 2023). We use the term epigenetics to refer to changes in gene expression patterns that are not caused by an underlying DNA sequence change, and that can be transmitted through cell division. The most common mechanisms that mediate epigenetic changes are DNA methylation, histone modification, and small RNAs (Kronholm, 2017). These mechanisms are capable of generating diverse phenotypes within an isogenic population by controlling gene expression patterns. Also, the reversible nature of these epigenetic mechanisms makes the genome flexible to respond to environmental changes (Sabarís et al., 2023). Transcription is controlled by transcription factors, and in the end they are responsible for regulation of transcription that happens during the lifetime of an organism (Davidson, 2006). Epigenetic mechanisms are one layer of regulation; for example, certain proteins can recognize DNA methylation and prevent the binding of transcription factors (Mattei et al., 2022). However, not all the epigenetic marks in the genome will have a transcriptional effect. What makes epigenetic regulation special is the transmission of the epigenetic states across cell division.

Mechanisms that mediate epigenetic changes, such as DNA methylation, likely originated as a defense mechanism against the proliferation of transposable elements or viral DNA within genomes, later their roles expanded to encompass various genome processes (Sánchez‐Romero & Casadesús, 2020). These mechanisms are present across the domains of life, but exhibit variation both within and across taxa. Particular epigenetic mechanisms are present in different taxa, and even each species can have its own mechanistic peculiarities (for examples in higher eukaryotes see Bewick et al., 2017; Klughammer et al., 2023). Broadly, prokaryotes rely on DNA methylation as an epigenetic mechanism, since they lack histones. However, they do have histone‐like proteins perform a comparable function (Carabetta, 2020; Sánchez‐Romero & Casadesús, 2020). In eukaryotic microbes, such as microscopic fungi, the epigenetic mechanisms are more diverse. Most of our mechanistic understanding comes from investigations in yeasts such as *Schizosaccharomyces pombe* and the filamentous fungus *Neurospora crassa*, for which the epigenetic machinery seems to mainly rely on histone modifications and small RNAs (Allshire & Selker, 2009).

In this review, we will explore research conducted on AMR within natural environments, with a specific focus on the epigenetic mechanisms that may confer resistance. Our examination will center on bacteria and microscopic fungi, as they are among the most common pathogens in humans, animals, and plants. Furthermore, considering the extensive utilization of antibiotics and antifungals, which currently stand as the primary contributors to the development of AMR in both clinical and natural environments (Fisher et al., 2022; Frieri et al., 2017; Lockhart et al., 2023), treating them together seems reasonable. Due to space constraints, we will not examine protists in detail, even if this class contains important pathogens that have evolved AMR, such as malaria.

## 2 ANTIMICROBIALS IN THE WILD

Antimicrobial substances have existed naturally in the wild for long periods in the evolutionary history of microbes. Microbes and other organisms produce them to outgrow competitors or to avoid parasites. For instance, arthropods can develop symbiotic interactions or generate their own antimicrobials to defend against antagonists (Janke et al., 2022; Kett et al., 2021). In various ant species, gland secretions inhibit the growth of entomopathogenic fungi (Dall et al., 2012). Both bee (*Apis mellifera*) and ant (*Pachicondyla gueldi*) venom contains peptides with potent antimicrobial properties effective against bacteria (Orivel et al., 2001). Also, numerous instances of arthropod‐fungal interactions have been documented; for examples refer to (Aanen et al., 2002; Holmes et al., 2016; Scott et al., 2008; Yek et al., 2012). For this reason, antimicrobial resistance mechanisms have been evolving in the wild long before the current era of antimicrobial resistance. Indeed, D'costa et al. (2011) identified antibiotic resistance genes in 30,000‐year‐old sediments from the Beringian permafrost. The existence of antibiotic resistance mechanisms for several thousand years in nature explains the swift emergence of antibiotic‐resistant strains in clinical settings, suggesting that selection imposed by new antibiotics acts on pre‐existing resistance mechanisms that have been present in the wild for millennia (D'costa et al., 2011).

Although antimicrobial substances occur naturally in the wild, anthropogenic activities have increased their prevalence in natural environments. Leakage into the environment is mainly through pharmaceutical spillover, through antimicrobial products used in plantations and livestock, and through human excretion (Allen et al., 2010; Hiltunen et al., 2017; Kraemer et al., 2019; Larsson & Flach, 2022; Wilkinson et al., 2022). Wastewater treatment plants can eliminate some, but not all antimicrobials from either water or the sludge (Larsson & Flach, 2022). As a result, these end up in rivers or lakes, and the sludge ends up in fields to be used as fertilizer. For example, Yuan et al. (2021) isolated enterobacteria from six environments: hospital, livestock manure, agricultural soil, forest soil, river sediment, and wastewater to understand the persistence of the resistant bacterial phenotype in nature. They found that strains isolated from the hospital and from livestock manure had the highest survival capability when exposed to mult‐idrug treatments. This was confirmed by PCR amplification of 12 β‐lactamase resistance genes, from which they found that the diversity of β‐lactamase genes was higher in the hospital. However, the highest abundance of β‐lactamase was found in wastewater, river sediments, and agricultural soil (Yuan et al., 2021).

**FIGURE 1:**
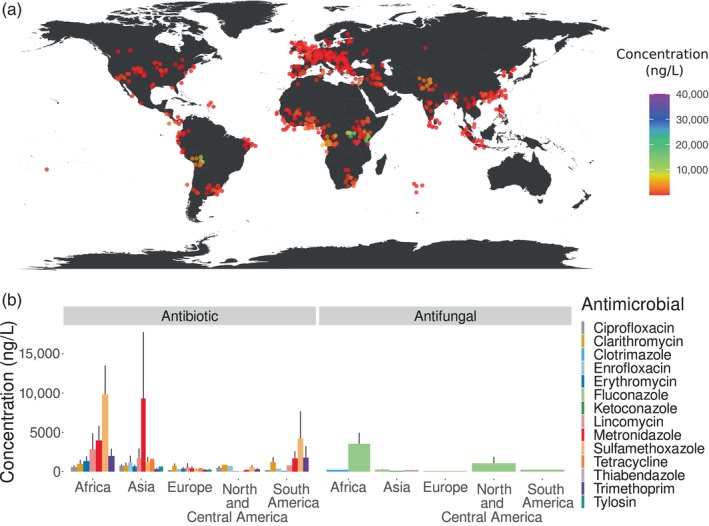
Global antimicrobial cumulative concentration. We show the sites and the antimicrobials that were detected according to data extracted from Wilkinson et al. (2022). Cumulative concentration is a general measure of the aquatic contamination. In this case is calculated as the sum of all the antimicrobial pharmaceuticals reported at all sampling locations in each continent (a). Sites where antimicrobial active ingredients were detected. We excluded the sites where antimicrobial concentration was zero (b). Concentration of each of the antimicrobial detected in each continent. Bars represent the standard error. Oceania is not included in panel b because antimicrobials were detected at a low concentration (80 ng/L), which is not clearly visible on the graph.

In a recent global study performed by Wilkinson et al. (2022), the authors reported the amount of 61 active pharmaceutical ingredients in 258 rivers in 104 countries. Of the 61 pharmaceuticals, 19 were antimicrobial (13 were antibiotics and 6 antifungals). Five of the 19 antimicrobials were not detected in any site (cloxacillin, sulfadiazine, oxytetracycline, itraconazole, and miconazole). Overall, the concentration of antibiotics was higher compared to antifungals. The study detected the highest concentration antimicrocbials in Africa, Asia, and South America. There was a negative relationship between country income and concentration of antimicrobials, countries with the lowest incomes had the highest concentrations. This may be associated with inefficient wastewater treatment infrastructure in developing countries (Wilkinson et al., 2022) (Figure 1). The lowest cumulative concentration was in Europe and North America and Oceania. In the latter, antimicrobials were only detected at one site. It is important to note that the samples were primarily collected from Europe; however, to the best of our knowledge, this study represents the most comprehensive global analysis with comparable measurements.

The study by Wilkinson et al. (2022) revealed that from the 13 of the antibiotics detected at least five (ciprofloxain, clarithromycin, lincomycin, trimethoprim, and metronidazole) exceeded the safe target concentration for AMR selection. In Africa and Asia, the concentration of all five of these antibiotics was higher than the target. In Europe and South America, four antibiotics exceeded the target, in North America only three while in Oceania only one. However, antibiotic concentrations in the environment are often orders of magnitude lower than the minimum inhibitory concentration (MIC) (Kraemer et al., 2019; Larsson & Flach, 2022). MIC is defined as the minimum amount of antibiotic needed to inhibit bacterial growth. It was previously assumed that antibiotic resistance was selected at a concentration above MIC (Kowalska‐Krochmal & Dudek‐Wicher, 2021). Untreated municipal sewage, treated sewage, rivers, and sea have been reported to contain antibiotic pollution at a concentration below 10 μg/L, which is below the typical MICs (10–10,000 μg/L). While industrially polluted surface water and untreated hospital effluent typically have antibiotic concentration above MIC (Larsson & Flach, 2022). For several years, a much debated question was whether low amounts of antimicrobials leaked into the environment could lead to antimicrobial resistance. However, there is now much evidence to suggest that sub‐inhibitory concentrations can select for antimicrobial resistance (Gullberg et al., 2011). First, it has been shown, using competition experiments, that bacterial strains that possess antibiotic resistant markers grow better than the wild‐type strain in antibiotic concentrations below the MIC, demonstrating that resistant strains have a selective advantage at low concentrations (Gullberg et al., 2011; Liu et al., 2011). Second, evolutionary theory suggests that it is easier to adapt to larger changes in the environment if the total environmental change occurs slowly or in small increments (Lindsey et al., 2013). Consequently, in laboratory settings, the probability that bacterial populations evolve antibiotic resistance is much higher if antibiotic concentration is slowly increased versus if the antibiotic concentration changes in a single step (Lindsey et al., 2013). Moreover, when the environment changed slowly, bacteria could evolve much higher levels of resistance as they acquired multiple mutations, compared to a single resistance mutation that was selected with abrupt environmental change (Lindsey et al., 2013). Moreover, further evidence shows that antibiotics can increase mutation rates, which in turn can increase the probability of resistance mutations (Gutierrez et al., 2013).

To date, a substantial body of evidence suggests that AMR in the wild is hugely influenced by human activity and presence. Wildlife populations closer to humans tend to exhibit greater persistence and higher levels of antimicrobial resistance compared to populations with little or no contact with human activity (Hwengwere et al., 2022; Skurnik et al., 2006; Sousa et al., 2014; Wellington et al., 2013). This phenomenon has been observed in various animal species, including penguins (Miller et al., 2009), gulls (Wallensten et al., 2011), other birds (Waldenström et al., 2005), the wild boar (Torres et al., 2020), and the Iberian lynx (Sousa et al., 2014). The results suggest that resistance in the wild is largely driven by human activity. For example, in Antarctica, samples were taken from water, sediment, and water‐filtering bivalves to isolate resistant strains (Hwengwere et al., 2022). Two types of bacteria were isolated: mesophiles, typically associated with humans, and psychrophiles, considered native to Antarctica. The study revealed that mesophiles exhibited higher resistance to a wider range of antibiotics compared to psychrophiles. Furthermore, resistance levels increased as sampling sites drew closer to research stations that represent more persistent human activity in Antarctica (Hwengwere et al., 2022). This finding is consistent with other studies that have identified a gradient of resistance levels in animals based on their proximity to humans. Samples taken from animals in close proximity to humans, including pets and domestic animals, showed the highest prevalence and levels of resistance compared to those from wild animals that reside further away from human populations (Skurnik et al., 2006). However, it is important to note that this pattern may differ in wild birds and other migratory species, as they can carry resistant strains to areas with low human activity (Wellington et al., 2013). For example, in remote locations such as Siberia, Alaska, and Greenland, the prevalence of resistance in birds exceeds that in local mammal populations (Radhouani et al., 2010; Wellington et al., 2013). Importantly, all of these studies demonstrate that direct use of antibiotics in the environment is not necessarily required to introduce resistant strains into wild populations. Instead, proximity to humans alone is sufficient for transmission (Hwengwere et al., 2022; Skurnik et al., 2006).

### 2.1 Epigenetic mechanisms for antimicrobial resistance

The genetic mechanism underlying AMR have been extensively studied. Some of these are innate, while others are acquired through gene transfer or via de novo beneficial mutations. However, these genetic mechanisms alone fail to fully explain all the processes through which microbes develop resistance. For example, the rapid emergence of resistance: survival rates observed when cells are exposed to antimicrobials are too high compared to what would be expected by genetic mutations alone. Adam et al. (2008) found that 20% of *E. coli* cells survive up to 1 μg/mL ampicillin, and this percentage is too high to be explained solely by the appearance of random genetic mutations. Even at reduced concentrations of antibiotics, the frequencies of genetic mutations are low and cannot account for the observed survival rate. For example, it is estimated that the probability of finding a genetic mutation that confers *P. aeruginosa* stable resistance to Quinolone is about $1.2\times 10^{-6}$ to $4\times 10^{-10}$ depending on the concentration used (Adam et al., 2008).

Hetero‐resistance is another instance that cannot be explained by genetic changes. Hetero‐resistance refers to the variable response to antimicrobial stress within an isogenic population (Ghosh et al., 2020). This heterogeneity can be explained by phase variation, which is the quick modification of gene expression patterns by switching on and off certain resistance genes. Phase variation is known to be regulated by non‐genetic mechanisms such as DNA methylation (Ghosh et al., 2020; Jiang et al., 2019). Phase variation is crucial for adaptive resistance, characterized by the temporary enhancement of microbes' ability to survive antimicrobial substances through alterations in gene expression (Hołówka & Zakrzewska‐Czerwińska, 2020; Veening et al., 2008). For example, evidence shows that adaptive resistance to certain antibiotics in *E. coli* is correlated with the amount of variation in the expression of the efflux pump system (Fernández & Hancock, 2012; Motta et al., 2015).

Adaptive resistance also includes the rapid emergence of the resistant phenotype and the capability to revert to the susceptible phenotype upon removal of antimicrobial stress (Ghosh et al., 2020). The restoration of the susceptible phenotype (Day, 2016; Ghosh et al., 2020) would necessitate a high number of back mutations, which are known to occur at a very low rate (Adam et al., 2008; Levin et al., 2000). For adaptive resistance to be efficient, heritable phenotypic variation mediated by epigenetic mechanisms should be transmitted across generations (Fernández & Hancock, 2012). Once antimicrobial stress is removed, the unstable epigenetic changes are no longer advantageous, eventually leading to the restoration of the original susceptible phenotype (Ghosh et al., 2020).

Antimicrobial resistance can also be explained by the appearance of persister cells. When a bacterial population is challenged with antibiotics, its population size will rapidly decline due to the high mortality rate. After the decline, the population size will stabilize and population growth slows down because the unaffected cells will enter a dormant state called persistence (Day, 2016). The persistence state is defined as a physiological state of dormancy that bacteria enter when they encounter environmental stressors (Day, 2016; Riber & Hansen, 2021), including low concentrations of antibiotics (Motta et al., 2015). This state is an effective strategy against insulting environments, as it provides resistance in conditions that would be harmful to normally growing cells. Interestingly, the persistence state is reversed in a drug‐free environment, where persister cells go back to a metabolically active state, reestablishing the original susceptible population (Day, 2016; Motta et al., 2015). This switch between active and dormancy states seems to be one of the main causes of adaptive resistance to antibiotic treatment, leading to high rates of bacterial infection relapses (Riber & Hansen, 2021).

Given this evidence, there is a growing realization that AMR largely depends on heritable phenotypic variation potentially caused by epigenetic changes. In the following section, we will describe the specific epigenetic mechanisms that have been associated with antibiotic resistance, emphasizing those that have been associated with below MIC adaptation and nonclinical environments.

### 2.2 Epigenetic mechanisms in bacteria and their role in antibiotic resistance

#### 2.2.1 DNA methylation

DNA methylation is the addition of a methyl group to cytosine or adenosine within DNA. Bacterial genomes can hold three types of DNA methylation: (1) 5‐methylcytosine (5mC), (2) N4‐methylcytosine (4mC), where a methyl group is added to either the position five or N4 of the cytosine, respectively, and (3) N6‐methyladenosine (6 mA), which is the most thoroughly studied (Sánchez‐Romero et al., 2020). This modification occurs when the N6‐position of adenosine is methylated (Wang et al., 2023). The enzymes responsible for all these modifications are DNA methyltransferases (MTases). MTases can be associated with the restriction‐modification (R‐M) defense system which is a defense against exogenous DNA. The DNA of the bacterial chromosome contains methylation, restriction enzymes recognize it and degrade only unmethylated DNA (Sánchez‐Romero & Casadesús, 2020). However, bacteria can also harbor MTases that are not part of the defense system that are known as orphan MTases. These orphan MTases perform essential functions within the cell, including influencing bacterial growth, participating in DNA repair processes, and regulating gene expression (Sánchez‐Romero & Casadesús, 2020).

Adenosine methylation occurs in bacteria mainly in palindromic sequences (5′‐GATC‐3′) (Sánchez‐Romero & Casadesús, 2020), but can occur in other motifs as well (Bruneaux et al., 2022). Palindromic motifs allow the inheritance of DNA methylation patterns. MTases recognize hemimethylated DNA formed after DNA replication and re‐methylate the unmethylated DNA strand. For adenosine methylation, the hemimethylated state is usually short‐lived, but stable hemimethylated GATC sites can form if a DNA‐binding protein blocks MTase activity. If this state persists through DNA replications, a non‐methylated site is formed. Such competition between MTases and DNA binding proteins is responsible for heritable epigenetic changes in bacteria, which are crucial for adaptive resistance to be effective (Hernday et al., 2003; Phillips et al., 2019). Such changes between methylated and non‐methylated states turn transcription off and on and affect many important phenotypes, such as pathogenicity, phage resistance, growth, antibiotic resistance, and gene expression heterogeneity (Atack et al., 2015; Cota et al., 2015; Sánchez‐Romero et al., 2020; Tram et al., 2021; van der Woude et al., 1996).

While the genome wide rates of spontaneous adenosine methylation changes are not known, some studies have looked at rates of adenosine methylation changes at individual loci. For example, Blyn et al. (1989) investigated the switching rate of the *pap*‐operon in *E. coli*. When *E. coli* were grown with glycerol as a carbon source, the rate of change from OFF to ON state was $1.57\times 10^{-4}$ per cell per generation, and rate from ON to OFF state was $2.60\times 10^{-2}$. When cells were grown with glucose as the carbon source, the rate of transition from OFF to ON state was $4.51\times 10^{-6}$. Glucose is a better carbon source than glycerol, so stress may increase rates of spontaneous adenosine methylation change. Moreover, we do know that switching events at different loci happen independently from each other (Sánchez‐Romero et al., 2020).

Empirical evidence has shown that various strains of *E. coli* mutants, lacking the MTase DNA adenine methylase, exhibited increased sensitivity and lower EC50 values when exposed to beta‐lactams, quinolones, and nalidixic acid. EC50 represents the antibiotic concentration that induces a biological response halfway between the baseline and the maximum response (Adam et al., 2008; Chen & Wang, 2021; Cohen et al., 2016). Together, all of this evidence suggests a crucial role for adenosine methylation in antibiotic resistance.

The effects of cytosine methylation in bacteria are not well characterized, although some evidence suggests that cytosine methylation is involved in transcriptional regulation during the stationary phase (Kahramanoglou et al., 2012). Furthermore, high levels of DNA cytosine methylation have been associated with antibiotic resistance. For example, studies have identified a positive correlation between 5mC and antibiotic resistance in enterobacteria (Yuan et al., 2021; Yugendran & Harish, 2016). Furthermore, DNA cytosine methylase knockout mutants of *E. coli* exhibited lower EC50 values when exposed to 20 different antibiotics (Chen & Wang, 2021). However, contradictory results have been reported in other studies in which *E. coli* mutants lacking 5mC did not show any significant effect on antibiotic survival (Adam et al., 2008).

Cytosine DNA methylation has been described to affect the regulation of the efflux pump system in *E. coli* and *Enterobacter clocae* (Fernández & Hancock, 2012; Militello et al., 2014). Efflux pump systems are energy‐dependent systems that allows the cell to expel toxic compounds from the inner‐cell environment to prevent the accumulation of toxins (Motta et al., 2015). Several efflux pump systems have been highly associated with antibiotic resistance; they have also been particularly characterized as being involved in multi‐drug resistance, as most efflux pump systems can transport more than one substance (Fernández & Hancock, 2012; Motta et al., 2015). For example, the membrane transporter *sugE*, which is classified as a multi‐drug resistance transporter has DNA cytosine methylase recognition sites in the gene body and upstream of the transcription start site (Militello et al., 2014). The influence of cytosine DNA methylation on *sugE* expression was confirmed by using DNA cytosine methylase knockout mutants. Knockout mutants express *sugE* at levels seven times higher compared to wild type, providing evidence that 5mC influences the sensitivity to an antimicrobial compound through changes in gene expression (Militello et al., 2014).

#### 2.2.2 Histone‐like proteins

The bacterial chromosome is circular and is not packed inside a nucleus around histones, as in eukaryotes. However, bacterial DNA still needs to be compacted. Bacteria pack their genomes around nucleoid‐associated proteins (NAPs) (Wang et al., 2023). NAPs are small proteins that fold and condense DNA and regulate gene expression. They participate in processes such as replication, translation, and repair of the bacterial genome (Amemiya et al., 2021; Stojkova et al., 2019; Wang et al., 2023). Also, under stress conditions, they can either help protect the DNA or induce transcriptomic changes to activate stress‐related genes (Amemiya et al., 2021; Hołówka & Zakrzewska‐Czerwińska, 2020).

NAPs differ between species, with those in *E. coli* being better described. The main NAPs are HU (heat‐unstable protein), IHF (integration host factor), H‐NS (histone‐like nucleoid structuring protein), Lrp (leucine‐responsive regulatory protein), Fis (factor for inversion stimulation), and Dps (DNA‐binding protein from starved cells) (Hołówka & Zakrzewska‐Czerwińska, 2020).

NAPs can undergo posttranslational modifications (e.g., acetylation or phosphorylation of lysines). These modifications influence DNA‐binding efficiency. Due to the crucial role that NAPs play in DNA condensation and stress response through phase variation, it is likely that NAPs and their chemical posttranslational modifications remain stable through several divisions (Ghosh et al., 2016). Additionally, protein posttranslational modifications have been proven to be a memory mechanism in bacteria (Lisman, 1985; Veening et al., 2008).

The first evidence for the role of NAPs in antibiotic resistance came from a study using *Mycobacterium smegmatis* (Sakatos et al., 2018). Previous studies with this bacterium had shown that subpopulations of persister cells would arise at a relatively high frequency within an isogenic population (Muhammad et al., 2022). Through transcriptomics and live cell imaging,c Sakatos et al. (2018), distinguished these persistent cells within a population and described their unique transcriptomic signatures. The persister phenotype was inherited by daughter cells and remained stable for short periods of time, although it was eventually lost in the absence of antibiotics. The authors were able to determine that the histone‐like protein HupB played a crucial role in the heterogeneous response, since deleting the HupB protein made the cell population more susceptible to antibiotics. Furthermore, they also discovered that mutating sites with post‐transcriptional modifications in HupB decreased the persister subpopulations (Sakatos et al., 2018). This study was the first to demonstrate that prokaryotes utilize posttranslational modifications to regulate antibiotic resistance.

Furthermore, studies in multi‐drug‐resistant bacteria *Acinetobacter baumannii* showed that the histone‐like nucleoid structuring protein (H‐NS) regulates the expression of genes involved in resistance to several antibiotics (Rodgers et al., 2021). Importantly, this histone‐like protein and other well‐known NAPs (IHF and HU) are crucial for biofilm formation. Biofilms are systems of microbial cells that are strongly associated with a surface embedded in a matrix of microbial origin (Dias et al., 2018). Biofilms highly enhance AMR in clinical and natural settings. In fact, more than 65% of microbial infections are caused by bacteria growing in biofilms (Dias et al., 2018; Wang et al., 2023). For example, wild proficient biofilm producers bacteria (*Acinetobacter* spp., *Klebsiella pneumoniae*, *Pseudomonas fluorescens*, and *Shewanella putrefaciens*) were highly resistant to multi‐drug treatments in their biofilm form (Dias et al., 2018).

#### 2.2.3 RNA modifications

The inheritance of factors such as RNAs and proteins during cell division has prompted the study of RNA molecule modifications as potential mechanisms for providing antibiotic resistance (AMR) across generations. In bacteria, various forms of methylation (5mC, 6 mA, and N1‐methyladenosine) have been identified on different types of RNA molecules, including transfer RNA (tRNA), messenger RNA (mRNA), ribosomal RNA (rRNA), and non‐coding RNA (ncRNA) (Marbaniang & Vogel, 2016; Shi et al., 2019). These modifications play a role in regulating and stabilizing RNA molecules, contributing to diversity in translation and creating rapid phenotypic variation (Evans et al., 2019).

Notably, rRNA methylation has been associated with antibiotic resistance by preventing antibiotics from binding to their target sites (Liu et al., 2015; Tada et al., 2013). Additionally, the knockout of tRNA methyltransferase affects the biosynthesis of the double membrane in gram‐negative bacteria, weakening the cell envelope structure, which serves as a permeability barrier and an anchor for efflux pumps (Hou et al., 2020; Masuda et al., 2019).

Recent research by Babosan et al. (2022), identified RNA modification genes not previously linked to antibiotic resistance as relevant for fitness in *Vibrio cholerae*. Particularly intriguing is the observation of these mechanisms in bacteria growing in sub‐minimal inhibitory concentration (sub‐MIC), making them especially pertinent for resistance in the wild. Transposon sequencing revealed differential activation or inactivation of genes under sub‐MIC antibiotic stress (tobramycin and ciprofloxacin), with RNA modification genes being enriched differently not only in the presence or absence of antibiotics but also when exposed to different antibiotics. This study sheds light on the broader role of RNA modifications in antibiotic resistance. Nevertheless, further research is essential, offering numerous opportunities for exploration in this field (Babosan et al., 2022).

Whether RNA modifications can be considered epigenetic remains to be seen. To be inherited, a particular type of RNA has to be stable enough to persist through cell division. For mRNAs this is unlikely to be true, as measured mRNA half‐lives in bacteria are around a few minutes (Selinger et al., 2003). Ribosomal RNA is likely stable enough as ribosomes are stable in growing bacterial cells (Piir et al., 2011). Moreover, the RNA modifications would likely have to be present in large enough numbers to have an effect, which would likely require that the modification is originally triggered by an environmental signal.

### 2.3 Epigenetic mechanisms for antimicrobial resistance in fungi

#### 2.3.1 Histone modifications

In eukaryotic cells, genomic DNA is packed in chromatin, which is made up of nucleosomes. Each nucleosome comprises approximately 146 base pairs of DNA wound around eight histone proteins, including two subunits each of the histones H2A, H2B, H3, and H4 (Freitag, 2017). These histones can have posttranslational modifications on certain residues on their N‐terminal tails, such as methylation, acetylation, phosphorylation, and ubiquitination. These modifications serve as key regulators of chromatin structure, by making DNA more or less accessible to the transcription and repair machinery (Freitag, 2017).

Most functional descriptions of fungal histone modifications come from model species, such as budding (*Saccharomyces cerevisiae*) and fission (*Schizosaccharomyces pombe*) yeast. The landscape of histone modifications among fungal species is generally well conserved. However, it is important to note that certain histone modifications are not present in all fungal species (Freitag, 2017). For example, SET enzymes, the proteins responsible for the methylation of the N‐terminal histone tails, are present among most, but not all fungal species. Budding yeast lacks methylation at lysine 9 of histone 3 (H3K9) (Freitag, 2017; O'Kane & Hyland, 2019), while it occurs in the fission yeast and several other filamentous fungi (e.g., *Mucor*, *Rhizopus*, and *Aspergillus*; Brosch et al., 2008). Furthermore, methylation on lysine 27 of histone 3 (H3K27) is absent in the budding yeast, fission yeast, and several filamentous fungi (Brosch et al., 2008; O'Kane & Hyland, 2019), but other filamentous fungi such as *Neurospora crassa* and *Fusarium graminearum* do exhibit methylation at this position (Brosch et al., 2008).

In fission yeast *Schizosaccharomyces pombe*, the mechanisms of epigenetic inheritance through histone modifications are best understood. Yeast geneticists have known for some time that *S. pombe* can exhibit a “culture memory,” where previous environmental conditions can affect the growth of a population (Petersen & Russell, 2016). The mechanism behind these effects can be the formation of heterochromatin and associated transcriptional changes. This process is guided by small RNAs (Yamanaka et al., 2013), indeed epigenetic inheritance has been demonstrated in fission yeast (Audergon et al., 2015; Ragunathan et al., 2014; Yu et al., 2018). The main silencing epigenetic mark in *S. pombe* is H3K9me and short interfering RNAs (siRNAs) which are required to maintain the silenced epigenetic state across cell divisions (Yu et al., 2018).

Chromatin rearrangements and histone modifications can facilitate the expression of different phenotypes, providing additional mechanisms through which organisms cope with antifungal drugs. For example, histone acetylation has been demonstrated to play a role in antifungal resistance in *Candida albicans* (Chang, Yadav, et al., 2019; Garnaud et al., 2016). Deacetylase proteins have been shown to be crucial in antifungal resistance. Genes encoding deacetylases, such as HDA1 and RPD3, exhibit higher expression levels in strains resistant to azoles (Garnaud et al., 2016). Furthermore, depletion of H3K56 acetylation leads to a reduction in virulence. This is also true for an alternative deacetylase complex composed of Set3, Hos2, SNT1, and Sif2, which mediate antifungal resistance in *C. albicans* biofilms (Nobile et al., 2014). Similarly, in *Cryptococcus neoformans*, the deletion of histone deacetylase genes weakens pathogenicity and affects sensitivity to various environmental stressors. Deacetylase proteins, in addition to their action on histones, can also regulate other proteins, including the heat shock protein 90, which is essential for stress response, virulence, and drug resistance (Lamoth et al., 2015).

It is important to note that even when two species share the same epigenetic pathways, their functions may differ. For example, methylation of lysine 4 in histone three (H3K4me) plays a crucial role in antifungal resistance in both budding yeast *Saccharomyces cerevisiae* and *Candida glabrata* (Baker et al., 2022). However, the antifungal resistance conferred by H3K4me may be attributed to the regulation of different pathways. In the case of budding yeast, the absence of H3K4 increases susceptibility to azoles by preventing overexpression of efflux pumps. These efflux pumps, similar to those described in bacteria, expel toxins from the cell. In the other case, the same epigenetic mechanism in *C. glabrata* increases susceptibility to azoles by affecting the expression of genes involved in the ergosterol biosynthesis pathway, which helps to maintain cell membrane integrity in fungi (Baker et al., 2022).

In fission yeast, it was shown that heterochromatin silencing, orchestrated by the H3K9me epimutation, can allow adaptation to caffeine (Torres‐Garcia et al., 2020). Yaseen et al. (2022) dissected the underlying molecular mechanism by which H3K9me confers resistance to caffeine. Exposure to caffeine affects the regulation of Epe1, which contains a conserved domain that promotes histone demethylation and is crucial for the formation of heterochromatin‐euchromatin boundaries (Sorida & Murakami, 2020). Exposure to environmental insults results in the accumulation of a truncated form of Epe1, which, in turn, increases H3K9me in several regions of the genome, reducing the expression of the underlying genes and improving resistance (Torres‐Garcia et al., 2020; Yaseen et al., 2022). This research is relevant because it untangles the molecular mechanisms behind resistance that is conferred exclusively by epigenetic mechanisms. Furthermore, caffeine‐resistant strains show cross‐resistance to antifungal agents. Similar heterochomatin silencing mechanisms are highly conserved in several pathogenic fungi, suggesting that similar silent mechanisms could be behind antifungal resistance also in the wild.

#### 2.3.2 RNA‐based mechanisms

The transcriptional products of non‐coding genes can be broadly classified as small non‐coding or long non‐coding RNAs (lncRNAs), both of these have been shown to impact antifungal resistance (Chang, Yadav, et al., 2019). Small interfering RNA molecules (siRNAs) are one of the best understood mechanisms of gene silencing in fungi. siRNAs are 20–30 base pair long RNA fragments that repress gene expression (Dang et al., 2011). *Mucor circinelloides* resistance to the FK506 antifungal agent is a well‐known examples of how epimutations can confer antimicrobial resistance via siRNAs (Chang, Yadav, et al., 2019). Calo et al. (2014) described that in *M. circinelloides*, endogenous expression of siRNAs conferred resistance by silencing the expression of the *fkbA* gene. *fkbA* encodes the substrate on which the FK506 antifungal acts. The authors also found that when the fungus was returned to a drug‐free environment, wild‐type resistance was restored as siRNA epimutation was no longer present. It was also described that this same fungus can use the same mechanism to gain resistance to the antifungal 5‐fluoroorotic acid (5‐FOA) by siRNAs (Chang, Billmyre, et al., 2019). The accumulation of siRNAs silences the expression of the *pyrF* or *pyrG* genes, which produce necessary enzymes that convert 5‐FOA into a toxic agent for the cell (Chang, Billmyre, et al., 2019; Chang, Yadav, et al., 2019).

In the fission yeast, it has been shown that lncRNAs can regulate antifungal resistance. Ard et al. (2014) demonstrated that by deleting the lncRNA ncRNA.1343, they could increase the sensitivity to a broad spectrum of antifungals. In this same investigation, they described that this is possible because ncRNA.1343 controls the regulation of a neighboring gene, tgp1, which encodes a glycerophosphodiester membrane transporter. The lncRNA increases nucleosome density, impeding transcription factor binding, resulting in the downregulation of tgp1. Finally, they demonstrated that the deletion of the lncRNA induces the expression of tgp1, demonstrating that ncRNA.1343 can regulate antimicrobial resistance in the fission yeast.

## 3 EDITING THE EPIGENOME

Due to the substantial body of evidence that points to the significant role of epigenetics in AMR, it becomes imperative to establish a causal relationship between epigenetic mechanisms and antimicrobial resistance. The comprehension and disentangling of complex regulatory systems regarding the relationship between epigenetics and AMR are vital for fundamental and applied research, offering promising avenues to combat the growing challenge of antimicrobial resistance.

The research reviewed here reveals a robust correlation between epigenetic states and antimicrobial resistance through gene regulation. However, demonstrating a direct causal relationship between epigenetic states and gene expression has proven to be a challenging task. Nevertheless, this could be made possible thanks to the availability of numerous epigenetic editing tools. Allegedly, nearly every locus in the genome can be targeted using these tools to modify expression patterns. Achieving this involves making site‐specific alterations in the epigenome through the use of programmable DNA‐binding domains (Thakore et al., 2016). Among these domains, zinc fingers, transcription activator‐like effectors (TALEs), and type II CRISPR are the most used (Thakore et al., 2016). These programmable DNA‐binding domains have been successfully utilized for targeted transcriptional activation and repression, providing evidence of causality, functionality, and cross‐talk among epigenetic marks. Just a few studies have effectively substantiated the causal relationship between epigenetic states and patterns of gene regulation (Policarpi et al., 2021).

The efficacy of epigenetic editing in the realm of antimicrobials remains a near‐term objective. However, there is a lack of research investigating epigenetic editing in microbes within the context of antimicrobial resistance. To date, the epigenetic editing advancements are experimental. While these tools continue to advance, questions regarding their specificity persist. Several studies have identified substantial off‐target effects associated with the three primary types of programmable DNA‐binding domains (Policarpi et al., 2021; Thakore et al., 2016). It is crucial to refine and improve these techniques, given that, in many cases, epigenetic editing is preferred over genetic editing. This preference relies on the reversible nature of epigenetic changes and their inducibility in specific tissues, developmental stages, or environmental conditions, often facilitated through the utilization of chemically inducible promoters (Thakore et al., 2016; Veley et al., 2023).

Epigenetic editing in host organisms has already demonstrated the potential of epigenetic editing in combating microbial infections. A notable example of this is the case of the cassava bacterial blight disease. Cassava is widely cultivated for numerous purposes, including human and animal consumption, the production of flour, alcohol, starches, sweeteners, and textiles, and it is susceptible to a disease caused by the bacterium *Xanthomonas phaseoli pv. manihotis* (Veley et al., 2023). Research has shown that the pathogenic bacteria uses the TAL20 (transcription activator‐like effector) to induce expression of the susceptibility gene MeSWEET10a, which belongs to the sugar transporter family. Activation of MeSWEET10a leads to observable symptoms, such as leaf lesions and potential plant death. Mutating MeSWEET10a is undesirable, as it plays a crucial role in normal plant development. Instead, Veley et al. (2023) conducted targeted methylation to the TAL20‐binding site within the MeSWEET10a promoter using a synthetic zinc‐finger DNA‐binding domain fused to a component of the RNA‐directed DNA methylation pathway. DNA methylation prevents the binding of the effector to the MeSWEET10a promoter, blocking transcriptional activation. Their findings demonstrated that this targeted methylation reduced the plant's symptoms without interfering with regular plant development. Furthermore, epigenetic editing has been applied to other crops to combat bacterial infections without the need for antibiotics (Selma & Orzáez, 2021).

## 4 EVOLUTION OF ANTIMICROBIAL RESISTANCE AND EPIGENETICS

### 4.1 Epigenetics and adaptation

Given that epigenetic variation is likely to be prevalent in microbes and can contribute to the evolution of antimicrobial resistance, what is the expected contribution of epigenetic changes? Hetero‐resistance and phase variation are key mechanisms by which epigenetic mechanisms confer AMR. These can be accomplished through epigenetic changes that switch specific genes ON and OFF, resulting in the emergence of resistant phenotypes governed by epigenetic mechanisms. However, these resistant traits are unstable, and the original antimicrobial susceptibility can be reinstated once the substance is withdrawn. Consequently, the significance of these epigenetically determined phenotypes in evolution has been widely debated. Nevertheless, maintaining the ability for epigenetic switching is advantageous depending on the environment and the rate of environmental changes. Studies indicate that phenotypic heterogeneity and stochastic transitions between phenotypic states are favored during adaptation to fluctuating environments (Stajic et al., 2022). This is highly relevant for natural microbial populations, given the variability in natural environments. In this context, epigenetic switching can be selected and become a fixed mechanism by which microbes deal with environmental fluctuations, as seen in the case of opvAB, a *Salmonella enterica* operon that undergoes bistable expression control through DNA methylation (Olivenza et al., 2019).

More generally, epigenetic changes are expected to speed up adaptation. This is because rates of spontaneous epigenetic changes are orders of magnitude faster than genetic mutation rates (Kronholm, 2017). Evolutionary models suggest that if it is possible to adapt via an epigenetic change, then the waiting time for the beneficial epigenetic change is going to be shorter than for a genetic mutation, and adaptation will initially proceed via using epigenetic changes (Kronholm & Collins, 2016). Since these epigenetic changes are also relatively unstable, they can lead to an evolutionary dynamic in which adaptation occurs initially with epigenetic changes, which are later replaced by more stable genetic mutations (Kronholm & Collins, 2016). This two‐phase dynamic has been observed in the lab with an artificial yeast system (Stajic et al., 2019). Epigenetic changes in microbes clearly fulfill this criteria, as many of such changes have been found when an excess number of resistant colonies grew on plates (Adam et al., 2008; Sakatos et al., 2018; Torres‐Garcia et al., 2020).

In addition to the mutation rate effect, epigenetic changes may indirectly help the evolution of resistance. It appears that many epigenetic changes that give antimicrobial resistance confer resistance to low levels of antimicrobials (Torres‐Garcia et al., 2020); therefore, it can be hypothesized that the low antimicrobial concentrations present in nature may select epigenetic changes that slightly improve fitness in the presence of the antimicrobial. If the population later encounters a higher dose of the antimicrobial, the mutational supply for genetic resistance mutations may increase. The mutational supply of resistance mutations, u=Nlμ, is the product of the number of chromosomes in the population, N for haploids, the number of loci in which resistance mutations can occur, l, and the mutation rate per locus, μ. Theoretically, epigenetic mutations can affect all three parameters, either population size, mutational target size, or mutation rate. Population size effect may occur if the epigenetic changes allow population size to remain higher for a longer period of time in the presence of high concentration of the antimicrobial, thus allowing for more chances for genetic resistance mutations to occur. Population size has been shown to have large effect on the probability of evolutionary rescue in experiments (Bell & Gonzalez, 2009).

Mutational target size effect may occur if the epigenetic change increases the number of genetic mutations that give resistance to the antimicrobial. This could happen, for example, by allowing for genetic mutations that together with the epigenetic change can make the cell resistant, either through additive effects or positive epistasis, but which alone would not be sufficient to rescue the population. It is known from theory and experiments that when a population adapts by smaller steps there are more mutational paths available (Lindsey et al., 2013).

The last possibility that epigenetic changes interact with the rate of genetic mutations is theoretically possible but perhaps unlikely. We know that epigenetic modifications interact with the mutation rate, for example, mutation rate is higher in densely packed heterochromatic regions that are marked by methylation of lysine 9 in histone 3 (Habig et al., 2021; Villalba de la Peña et al., 2023). Furthermore, methylated cytosines are more susceptible to deamination than unmethylated cytosines (Zhang & Mathews, 1994), and methylation may also affect the probability of mutation in the surrounding bases (Kusmartsev et al., 2020). Therefore, if antimicrobial resistance depends on silencing a particular gene, an epigenetic change that increases the probability of genetic mutations can in principle increase the supply of resistance mutations. However, this scenario requires resistance to be given by silencing a particular gene.

At the moment, the indirect ways described above, in which epigenetic variation can increase the rate of adaptation on top of the increased mutation rate from epigenetic changes themselves, represent a hypothesis. We suggest that these are interesting avenues for future research.

### 4.2 Priming

Epigenetic mechanisms sometimes also mediate a phenomenon called priming (Harish et al., 2022). While the phenomenon of priming may in some cases caused by nonepigenetic mechanisms, we will discuss priming here, since priming can affect the dynamics of resistance evolution. The phenomenon of priming happens when low concentrations of antimicrobials may also induce a general stress response that allows the next generation to tolerate stressful conditions, such as a higher concentration of antimicrobials. This effect has been referred to by multiple names in addition to priming in the literature, such as acquired stress response. In the ecological literature, similar phenomena are called a parental effect or phenotypic plasticity. Priming is a deterministic response, and this phenomenon has been observed for a diverse set of traits in fungi and bacteria, reviewed by Harish and Osherov (2022). Priming may be intra‐, inter‐, or transgenerational. In the fungus *Aspergillus fumigatus*, low concentration of fungicide primed the next generation to be able to tolerate a much higher concentration and increased the probability of developing resistance (Harish et al., 2022). In this case, the mechanism is currently unknown. Another example of fungal priming comes from the pathogenic yeast *Candida albicans*, where a glucose‐induced stress response gave resistance to a fungicide (Rodaki et al., 2009).

Priming has also been observed to give antibiotic resistance in bacteria. Sublethal concentrations of ampicillin have been found to prime *E. coli* cells for general stress tolerance that includes increased tolerance to antibiotics (Mathieu et al., 2016). Sublethal concentration of antimicrobial peptides can also induce a similar response in *E. coli* (Rodríguez‐Rojas et al., 2021).

Priming will influence subsequent evolutionary adaptation in the same way as phenotypic plasticity. It will bring the population closer to the fitness optimum, thus making the amount the population needs to adapt smaller, speeding up adaptation. This is also called the Baldwin effect (Kronholm, 2017). Plasticity can also speed up adaptation by keeping the population size large, thus increasing the mutational supply (Chevin et al., 2010).

Furthermore, there is another hypothesis on how phenotypic plasticity, or priming, could also facilitate adaptation. Theoretical models suggest that plasticity can align mutational and genetic variance along the axis of environmental variation (Draghi & Whitlock, 2012). For example, if an organism contains the genetic machinery to be phenotypically plastic in response to a certain environmental variable, it has a larger mutational target size for changing this phenotype. The empirical evidence for this hypothesis is mixed, with some support (Lind et al., 2015) and some contradicting results (Johansson et al., 2023). Testing this hypothesis would be interesting in the context of priming and antimicrobial resistance.

## 5 CONCLUSION

In conclusion, the widespread use of antimicrobial drugs has resulted in an alarming infiltration of these agents and antimicrobial‐resistant strains into natural environments. This infiltration poses a significant threat to overall environmental health. Even antimicrobials at sub‐inhibitory concentrations in the wild represent a concerning issue, as they can still substantially increase the risk of resistance development. Evidence has demonstrated that in microbes, epigenetic mechanisms are crucial for developing antibiotic resistance, particularly via hetero‐resistance and phase variation. Also, epigenetic mutations are also crucial in adaptation, particularly to low antibiotic doses. Thus, continuing to investigate the potential of epigenetic changes to increasing antimicrobial resistance in the wild is crucial.

Significant knowledge gaps persist on the contribution of epigenetic changes to the evolution of antimicrobial resistance. Key questions remain unanswered: Does epigenetic variation expedite resistance evolution in natural settings and does it enhance resistance probabilities directly or indirectly? Addressing these questions requires thorough observations and empirical studies. Previously, methodological challenges hindered the study of epigenetic effects in microbes. However, recent advances in sequencing technologies and epigenetic editing methods offer promising avenues for rigorously addressing this questions and testing causal relationships. Given the amount of knowledge accumulated thus far and the technology available, we are optimistic that answers to these questions are now within our reach.

## CONFLICT OF INTEREST STATEMENT

The authors declare no competing interests.

## Data Availability

The data used in this paper is deposited in Dryad (https://doi.org/10.5061/dryad.j9kd51ckm).
